# Adherence to medication and gender are associated with uncontrolled hypertension: findings of a cross-sectional study in East-Central Uganda

**DOI:** 10.11604/pamj.2026.53.127.45349

**Published:** 2026-03-13

**Authors:** Nicholas Higenyi, Victoria Nankabirwa, Philip Ajuk

**Affiliations:** 1 Department of Family Medicine, School of Medicine, College of Health Sciences, Makerere University, Kampala, Uganda,; 2 School of Public Health, College of Health Sciences, Makerere University, Kampala, Uganda

**Keywords:** Uncontrolled hypertension, East-Central Uganda, adherence to medication, determinants

## Abstract

**Introduction:**

while uncontrolled hypertension (UCH) affects mostly developing countries, there is limited information on its burden and associated factors in Uganda. This study, therefore, sought to determine its prevalence and associated factors in East-Central Uganda.

**Methods:**

we used systematic sampling to enroll 318 clients living with hypertension, aged 18 to 69 years, attending Kamuli General Hospital, for this cross-sectional study. A pretested structured questionnaire adapted from the World Health Organization (WHO) STEPS tool was used to collect the study data as participants exited the clinic. The participants´ blood pressure (BP) and anthropometric measurements were taken as per the WHO STEPS manual. Uncontrolled hypertension was defined as an average systolic blood pressure of at least 140 mmHg and/or an average diastolic blood pressure of at least 90 mmHg. Robust Poisson regression was used for multivariable analysis of the relationship between UCH and the other study variables.

**Results:**

of the 318 participants enrolled, 289 had complete information and were included in the analysis. The majority (79.6%) were females aged at least 51 years of age (58.2%). The median and mean age were 53 years (Q1 = 45.0, Q3 = 60.0), with a standard deviation of 9.6 years. The mean SBP and DBP were 162.4 ± 22.8 mmHg and 97.5 ± 15.7mmHg respectively. The prevalence of UCH was 85.8% (95% CI 81.3% - 89.4%). Adherence to antihypertensive treatment and gender were strongly associated with UCH. The prevalence of UCH did not vary significantly by level of education, level of physical activity, or history of use of herbal medication among the clients.

**Conclusion:**

the prevalence of UCH was very high, requiring urgent interventions prioritizing male hypertensive patients and approaches aimed at improving adherence to antihypertensive treatment.

## Introduction

Uncontrolled hypertension is the major risk factor for cardiovascular disease and mortality in both low- and high-income countries [1]. Persistently uncontrolled hypertension increases the risk for long-term complications such as myocardial infarction, heart failure, stroke, and kidney disease [2]. The reasons for the poor control majority of hypertension vary from health system related factors to individual and behavioral factors in China, a study showed that being elderly, living in rural and suburban areas, having low education level, having a family history of high blood pressure, smoking cigarettes, consuming excess salt, having a sedentary life style, obesity and having diabetes mellitus were associated with UCH [3]. Studies in Sudan [4], Tanzania [5], Cameroon [6], and Nepal [7] found no association between sociodemographic characteristics (such as: age, sex, and alcohol) and UCH. In Uganda, the majority of people with hypertension are unaware, and even among those who were aware, less than half were receiving treatment [8]. Effective control of hypertension also depends on accessibility and the quality of the healthcare system [9]. However, in Uganda and most of sub-Saharan Africa, the health care system still faces sub-optimal levels of health staffing, frequent stock- outs of antihypertensive medication, low client literacy levels, making achieving adequate control of hypertension an uphill task [8,10]. The world hypertension league (WHL) recommends that community organizations develop high-capacity BP screening programs that connect persons with high readings to health care as well as for regulations to ensure the use of accurate and appropriate BP devices and cuffs [11]. Also, the American Heart Association (AHA) and the National Institute for Health and Care Excellence (NICE) recommend physical exercise as adjuvant treatment for hypertension. Community based and lifestyle-linked interventions, such as restricted salt in food, control of overweight and obesity, potassium-rich diet of fresh fruits and vegetables, reduction of alcohol consumption and cessation of cigarette smoking are also recommended for the control of hypertension [12]. The extent to which these recommendations were adhered to at the individual level in East Central Uganda was not known. This study therefore sought to determine the prevalence of uncontrolled hypertension and associated factors among clients living with hypertension attending Kamuli General Hospital, East-Central Uganda.

## Methods

**Study design and setting:** this cross-sectional study conducted at Kamuli General Hospital, in East Central Uganda, between January 2021 and September 2021, was approved by the Makerere University College of Health Sciences, School of Public Health, Higher Degrees Research and Ethics committee on 13^th^ November 2020. The study population was adult patients living with hypertension in the district aged at least 18 years. The rationale was that the majority of patients living with hypertension fell in this age category.

**Study population and eligibility:** a total of 289 participants were included in the study by using the Kish-Leslie formula. A 95% confidence interval and taking 5% level of precision. The prevalence of uncontrolled hypertension in a study in 2014 by the Ministry of Health was found to be 75% [13]. Systematic random sampling was done, with an interval of 3 used. All the hypertensive patients between 18 and 69 years of age who attended the clinic during the time of the study were eligible for inclusion in the study while the critically ill and those with cognitive impairment were excluded from the study.

**Data collection procedure and tools:** data was collected using an interviewer-administered structured questionnaire adapted from the WHO STEPS tool. The tool was adapted for the study by deleting the cervical cancer screening section and step 3 of the instrument, which required biochemical measurements. Also, locally available fruits and vegetables were included in the tool as examples. The questionnaire also assessed medication adherence using the 4-item Morisky medication adherence scale. Alcohol consumption was assessed using locally accessible brands of alcohol to suit the local context.

### Study variables

**Dependent variable:** the main study outcome variable was hypertension control status, which took on two values: uncontrolled hypertension later coded as 1, and controlled hypertension, later coded as 0. Uncontrolled hypertension was defined as having average systolic blood pressure ≥ 140 mmHg and/or diastolic blood pressure ≥ 90 mmHg in patients taking anti-hypertensive treatment.

**Independent variables:** independent variables included: age, gender, monthly income, level of education, and comorbidity status. The other independent variables that were evaluated included: level of physical activity, salt consumption, sedentary behavior, monthly household income, body mass index, health education advice, presence of comorbidities, antihypertensive medication adherence, diet, use of traditional/herbal medication, alcohol intake and cigarette/tobacco smoking. Medication adherence was assessed using the 4-item Morisky medication adherence scale. The scale has been reported to have good internal consistency with Cronbach´s alpha of 0.90 in some studies of patients with hypertension [14]. Patients responded “yes” or “no” to the four questions in the scale. ‘’Yes’’ responses were scored as 1, and ‘’no’’ responses were scored as 0. The adherence score was the total sum of the scores of the responses. Based on the scores, individuals who had a total score of zero were classified as adherent and those with a total score of at least 1 were classified as non-adherent to antihypertensive treatment. Diet Respondents´ diet was assessed by collecting data on the frequency of consumption of the locally available fruits and vegetables in a typical week. Photographic aids were provided, and participants responded to how frequently they consumed the listed fruits and vegetables in a typical week. Physical activity was assessed in terms: work, travel to and from places and recreation activities.

The respondents were assessed on frequency, duration and intensity of physical activity. Metabolic equivalents (METS) were calculated by multiplying the number of minutes spent on vigorous activities by 8.0, moderate intensity activities by 4. The total weekly metabolic equivalents were derived by adding participants´ metabolic equivalents from vigorous intensity physical activities to metabolic equivalents from moderate intensity physical activities. Using the total number of METS per week, participants were classified as having adequate physical activity if their total weekly metabolic equivalents were at least 3000 otherwise they were classified as having inadequate physical activity as per the WHO global recommendations on physical activities for health [15]. Participants´ use of tobacco was assessed from current or history of use, the type and method of tobacco use (chewing, pipe or cigarette smoking). Alcohol use was assessed by collecting information on frequency, type and quantity of alcohol consumed. Respondents who consumed an equivalent of 60 grams (six standard drinks) of alcohol either regularly or in a binge were classified as having harmful alcohol use. Anthropometric evaluations including measurement of weight and height were taken by the trained research assistants. Weight was measured using a pre-calibrated weighing scale with participants wearing light clothing and barefooted. Weight was rounded off to the nearest 1 kg. Height was measured with the participant standing upright against a wall using an adult size stadiometer. Body mass index (BMI) of each participant was then derived by dividing the weight in kilograms by the square of the height in meters. Participants who had BMI of less than 25 kg/m^2^ were classified as having normal BMI whereas those with BMI of at least 25kg/m^2^ were classified as being overweight or obese.

**Sociodemographic data collected:** sex and age in completed years, number of years completed in school and the highest level of education attained by the participant. The highest level of education attained was dichotomized into utmost primary which included clients who attained only primary level education or less, and at least secondary category which included clients who attained secondary level education or more. Data was collected about the clients´ marital status. The marital status was later dichotomized into married for clients who lived with a partner and unmarried for clients who did not live with a partner. The clients were also interviewed about average annual and monthly household income and occupation.

**Statistical analysis:** data collected was entered using Microsoft Excel 2007 and exported to STATA 14 for analysis. Variables were summarized using frequencies, means with standard deviations or median, and interquartile ranges where appropriate. Pearson´s Chi-square and Fisher´s exact tests were used to determine the relationship between uncontrolled hypertension and other variables at bivariable level. Variables associated with uncontrolled hypertension at bivariable level with a P-value of 0.2 or less were considered for multivariable analysis using modified Poisson regression.

**Ethics approval and consent to participate:** ethical approval to conduct the study was granted by the Makerere University College of Health Sciences, School of Public Health Higher Degrees Research and Ethics committee on 13^th^/November/2020. Before participants’ recruitment in the study, informed consent was obtained from all participants.

## Results

**Characteristics of study participants living with hypertension at Kamuli General Hospital:** of the 318 participants enrolled in the study, 289 had complete records and were included in the analysis (25 participants did not have records of blood pressure taken at the preceding visit, while 4 participants needed emergency admission and treatment); Table 1 shows their sociodemographic characteristics. The majority of the participants (79.6%) were female and aged at least 51 years (58.2%). The median and mean age were 53 years (Inter quartile range=15), with a standard deviation of 9.6 years. The overall mean body mass index (BMI) was 27.5 ± 5.0 kg/m^2^. Among males, the mean BMI was 24.4 ± 3.8 kg/m^2^ and 28.3±5.0 among females. There was a statistically significant difference in the BMI between males and females. One hundred and seventy-seven (61.2%) of the participants were either overweight or obese. The proportion of overweight and obese female participants (69.1%) was more than double that of overweight and obese male participants (30.5%). The overall mean systolic blood pressure (SBP) was 162.4 ± 22.8 mmHg, with a mean of 164.2 ± 21.2 mmHg and 161.9 ± 23.2 mmHg among males and females, respectively. The overall mean diastolic blood pressure was 97.5 ± 15.7mmHg with a mean of 97.5 ± 14.9.0 and 97.5 ± 16.0 mmHg among males and females, respectively.

**Table 1:** socio-demographic characteristics of hypertensive clients attending Kamuli General Hospital

Variable	Hypertension state		Total
	Uncontrolled, N(%) 248(85.8)	Controlled, N(%) 41(14.2)	N 289
**Education level attained**			
None/Primary	159 (88.3)	21 (11.7)	180
Secondary and over	89 (81.7)	20(18.3)	109
**Employment**			
Unemployed/Retired	21 (84.0)	4 (16.0)	25
Self/employed	227 (86.0)	37 (14.0)	264
**Monthly household income (Ug. Shs)**			
Less than 350000	162(85.7)	27 (14.3)	189
At least 350000	86 (86.0)	14 (14.0)	100
**Age in years**			
18-35	8 (88.9)	1 (11.1)	9
36-45	54(81.8)	12 (18.2)	66
46-55	69 (84.1)	13 (15.9)	82
56-69	117 (88.6)	15 (11.4)	132
**Marital status**			
Married	216 (85.4)	37(14.6)	253
Unmarried	32(88.9)	4 (11.1)	36
**Sex**			
Female	192(83.5)	38 (16.5)	230
Male	56 (94.9)	3 (5.1)	59

**Prevalence of uncontrolled hypertension:** two hundred and forty-eight (85.8%) of the participants had uncontrolled hypertension. The prevalence of uncontrolled hypertension among patients living with hypertension attending Kamuli General Hospital was 85.8% (95% CI 81.3% - 89.4%). The prevalence of uncontrolled hypertension was 83.5% (95% CI 78.5%-88.3%) among females and 95.9% (95% CI 89.3% 100%) among male patients living with hypertension. The prevalence of uncontrolled hypertension was significantly different among male and female participants (p= 0.02).

**Factors associated with uncontrolled hypertension:** one hundred and eighty-nine (65.4%) of participants reported a monthly household income of less than 350000 Uganda shillings. The prevalence of uncontrolled hypertension among participants having a monthly household income of less than 350000 Uganda shillings was 85.7% (95% CI 80.7%-90.7%). This was not significantly different from the prevalence of uncontrolled hypertension among participants reporting a monthly household income above 350000 Uganda shillings (86 .0%) (95% CI 79.2%- 92.8%) p = 0.95). One participant (0.4%) reported use of tobacco, and she had uncontrolled hypertension. Five participants (1.7%) reported taking at least one standard drink of alcohol per week. None of the participants admitted binge drinking or had harmful use of alcohol. All the participants who reported a history of alcohol consumption had uncontrolled hypertension. All the participants reported that they had received lifestyle adjustment advice for the control of hypertension. There was no difference in the prevalence of uncontrolled hypertension across the seven age categories of the study participants (Pearson chi 2 = 2.2932 p = 0.89). Seventy-nine (27.3%) of the participants had been diagnosed with diabetes mellitus. Fifteen (5.2%) of the participants were diabetes mellitus patients on insulin. The prevalence of uncontrolled hypertension was marginally higher among diabetic participants (87.3% (95% CI 80.0% - 94.7%)) as compared to participants without diabetes (85.2% (95% CI 80.4-90.0%)). However, the difference in prevalence among diabetic and non-diabetic participants was not statistically significant (p= 0.65).

For self- reported salt consumption, 61(21.1%) of the participants reported consuming too little salt while the rest reported consuming just enough salt. The prevalence of uncontrolled hypertension was not statistically significantly (at 95% level) different for the two categories of salt consumption (p= 0.07). None of the participants had used herbal medication for the control of hypertension. The majority (89.6%) of the participants were involved in physical activities of at least 3000 METs per week. The most commonly practiced physical activity was gardening/digging. There was no statistically significant difference in the prevalence of uncontrolled hypertension between individuals who reported adequate physical activity and those who did not have adequate weekly physical activity. The bivariable analysis of factors associated with uncontrolled hypertension is shown in Table 2. The factors associated with uncontrolled hypertension after bivariable analysis were: adherence to antihypertensive treatment, and participant´s sex. Highest level of education attained, self- reported salt consumption, level of physical activity, monthly household income, sedentary behavior, body mass index and having a comorbidity of diabetes mellitus were not associated with uncontrolled hypertension at the 95% level of significance.

**Table 2:** bivariable analysis of factors associated with uncontrolled hypertension among hypertensive clients attending Kamuli General Hospital

Factor	Uncontrolled hypertension		Chi-square/Fisher's exact	P-value
	Yes	No		
**Adherence to medication**				
Non adherent	232	4	0.00	0.00*
Adherent	16	37		
**Sex**				
Female	192	38	0.02	0.02*
Male	56	3		
**Monthly household income**				
Less than 350000	162	27	0.00	0.95
At least 350000	86	14		
**Comorbidity-diabetes mellitus**				
Non diabetic	179	31	0.21	0.65
Diabetes comorbidity	69	10		
**Salt consumption**				
Too little	48	13	3.22	0.07
Just the right amount	200	28		
**Sedentary behavior**				
Less than 5 hours	164	32	2.29	0.13
At least 5 hours	84	9		
**Body mass index**				
Normal	94	18	0.53	0.47
Overweight/obese	154	23		
**Level of education**				
Primary or none	159	21	2.49	0.12
At least secondary	89	20		
**Alcohol consumption**				
Yes	5	0	1.00	0.46
No	243	41		
**Physical activity**				
Inadequate	27	3	0.78	0.36
Adequate	221	38		
**Cigarette smoking**				
Yes	1	0	1.00	0.86
No	247	41		
**Age group**				
18-49 years	100	21	1.72	0.19
50-69 years	148	20		
**Living with a partner**				
Yes	217	37	0.80	0.42
No	31	4		
**Use of herbal medication**				
Yes	1	0	1.00	0.86
No	247	41		
**Consumption of fruits and vegetables**				
Less than 7 servings/week	122	20	0.00	0.96
At least 7 servings/week	126	21		

Key * Factors significantly associated with uncontrolled hypertension after multivariable analysis

After multivariable analysis, the factors associated with uncontrolled hypertension were gender (aPR 1.12 (95% CI: 1.03-1.21), p=0.01) and adherence to antihypertensive treatment (aPR 0.31 (95% CI: 0.21-0.47), p<0.001)). When adjusted for highest level of education, sedentary behavior, self-reported salt consumption, and adherence to antihypertensive treatment, the prevalence of uncontrolled hypertension among male patients living with hypertension was 1.12 times that of female participants. Also, when adjusted for highest level of education, sedentary behavior, self-reported salt consumption, sex, the prevalence of uncontrolled hypertension among participants who were not adherent to antihypertensive treatment was 3 times that of participants who were adherent to antihypertensive treatment. There was no significant difference in the prevalence of uncontrolled hypertension among participants who reported taking just the right amount of salt and those who took too little when adjusted for gender, adherence to antihypertensive treatment, sedentary behavior and highest level of education attained. When adjusted for highest level of education, self-reported salt consumption, adherence to antihypertensive treatment and gender, there was no significant difference in the prevalence of uncontrolled hypertension among patients living with hypertension who reported less than 5 hours of sedentary behavior and those who reported at least 5 hours of sedentary behavior. There was no significant difference in the prevalence of uncontrolled hypertension among participants who attained utmost primary education as compared to those who attained at least secondary education when adjusted for gender, self-reported salt consumption, adherence to antihypertensive treatment and sedentary behavior (Table 3).

**Table 3:** multivariable analysis of factors associated with uncontrolled hypertension among hypertensive clients at KGH using modified Poisson regression

Factor	Uncontrolled hypertension		Adjusted PR	95% CI	P>|z|
	Yes	No			
**Adherence to medication**					
Not adherent	232	4	1.00R	1.00	
Adherent	16	37	0.31	0.21-0.47	0.00*
**Sex**					
Female	192	38	1.00R	1.00	
Male	56	3	1.12	1.03-1.21	0.01*
**Salt consumption**					
Too little	48	13	1.00R	1.00	
Just the right amount	200	28	1.07	0.97-1.17	0.18
**Sedentary behavior**					
Less than 5 hrs	164	32	1.00R	1.00	
At least 5 hrs	84	9	1.04	0.97-1.11	0.26
**Level of education**					
At least secondary	89	20	1.00R	1.00	
Primary or none	159	21	0.98	0.89-1.06	0.58
**Age**					
18-50yrs	100	21	1.00R	1.00	
51-69yrs	48	20	0.99	0.81-1.02	0.84

KEY * Factors significantly associated with uncontrolled hypertension after multivariable analysis

## Discussion

From this cross-sectional study based at Kamuli General Hospital among patients living with hypertension aged between 18 and 69 years, the prevalence of uncontrolled hypertension was 85.8%. The high prevalence of uncontrolled hypertension in this study is similar to findings from other studies done elsewhere in Uganda. In a study conducted in central Uganda in the districts of Mukono and Buikwe on prevalence, awareness, and control of hypertension in Uganda, the prevalence of uncontrolled hypertension was 90.6% [8]. Similarly, the prevalence of uncontrolled hypertension was 82.52% in a hospital-based study in South Western Uganda among patients with type 2 diabetes mellitus [16]. The high prevalence may be due to poor adherence to medication, which is mainly determined by access to antihypertensive medication, especially for the poorer patients [10]. Also, the high prevalence is comparable to findings from other studies done in 10 Eastern sub-Saharan African countries, where the level of uncontrolled hypertension was 88.4% [17]. It is also similar to the finding of a prevalence of 84.4% among the patients living with hypertension in the primary health care facilities of Kinshasa in the Democratic Republic of Congo [18]. The prevalence of uncontrolled hypertension is, however, much higher than findings in Sudan of 45.3% [6], South West Ethiopia of 49.7% [19], and North West Ethiopia of 53.4% [20]. The differences in prevalence of uncontrolled hypertension from that of other countries in sub-Saharan Africa may point to underlying differences in policies, socio-demographic characteristics of the hypertensive populations, and access to antihypertensive care.

**Factors associated with uncontrolled hypertension**: in this study, there was a statistically significant association between gender and uncontrolled hypertension, with the prevalence being higher among males as compared to females. Male participants were 12% more likely to have uncontrolled hypertension as compared to female participants. This finding is consistent with the findings of studies in Central Uganda [8], Morocco [21], and South Africa [22], where male gender was associated with uncontrolled hypertension. A possible explanation is that men generally tend to be poorer seekers of health care as compared to women and only tend to seek care when they develop symptoms or complications of a disease or health condition. The findings are different from those of a study in Yaounde, Cameroon [6], where there was no significant difference in the prevalence of hypertension among men and women living with hypertension. This may be due to the fact that the study was in an urban setting where women tend to assume gender roles similar to those of men and therefore pay less attention to their own health care needs as compared to semi-urban and rural women. Adherence to antihypertensive treatment was very strongly associated with uncontrolled hypertension. However, adherence to medication was very low (18.3%). A plausible explanation is that the majority of the participants reported skipping medication often when they felt well while other participants only took medication as long as the pills supplied during the clinic visits lasted. The prevalence of uncontrolled hypertension among participants who did not adhere to antihypertension treatment was more than triple that of patients who adhered to antihypertension treatment. The finding is consistent with the results of studies conducted in South Africa [22], Ghana [23], Ethiopia [24], Sudan [4], Tanzania [5], and Kenya [25] where uncontrolled hypertension was similarly associated with adherence to antihypertension treatment.

There was no statistically significant association between reported monthly household incomes and uncontrolled hypertension. The study findings are consistent with the results of a study in Ghana [23] and Ethiopia [24] where monthly household income was similarly not associated with uncontrolled hypertension. This differs from the study in Zimbabwe [26] where those who had a monthly household income of at least 200 USA dollars (about 700000 Uganda Shillings) were less likely to have uncontrolled hypertension. This is probably because patients living with hypertension who attend public general hospitals like the one where this study was conducted tend to fall in the lower income category, and hence, the household incomes did not differ widely across the study population. On the other hand, the study in Lupane Zimbabwe, included patients attending private health care who tend to fall in the higher income categories and could therefore afford better care. Also, there was no statistically significant association between level of education, age, hours of sedentary activity, and uncontrolled hypertension. This is consistent with the findings of the study of factors associated with blood pressure control amongst adults with hypertension in Yaoundé, Cameroon [6] and Sudan [4] where sociodemographic factors were not associated with uncontrolled hypertension. The findings differ from those of a study in China [3] where being elderly, low education level, excessive salt consumption, lack of physical activity, being overweight or obese, and having diabetes mellitus were associated with uncontrolled hypertension.

**The study may have had the following limitations:** self-reporting being used as the only method of measuring adherence has the disadvantages of recall bias and eliciting only socially acceptable responses, which may have overestimated the level of adherence. Recall and social desirability bias also apply to self-reported data on cigarette smoking and alcohol consumption. Another limitation of the study is that blood pressure measurements taken during clinic visits may be significantly different from those when participants are in their home environment due to the “white coat hypertension effect”. This may have resulted in overestimation of uncontrolled hypertension.

## Conclusion

The prevalence of uncontrolled hypertension among patients living with hypertension attending care at Kamuli General Hospital was very high. The best intervention areas will be improving adherence to antihypertensive medication and prioritizing the male hypertensive clients.

### What is known about this topic

- *Uncontrolled hypertension is a principal contributor to cardiovascular morbidity and mortality;*
- It is the most frequent cause of heart disease and cerebrovascular accidents including stroke;
- Reducing proportion of hypertensive patients with uncontrolled hypertension significantly reduces the frequency of stroke and heart disease in the population.

### What this study adds

- The study helps to quantify the burden of uncontrolled hypertension in the region;
- The study also points to the intervention areas where control efforts should focus; promoting adherence to antihypertensive medication and the male hypertensive clients.
